# Development and Consumer Acceptance of Low-Sugar Gummy Candies Naturally Colored with Sorghum Leaf Polyphenols

**DOI:** 10.3390/foods15173037

**Published:** 2026-08-28

**Authors:** Jaymi Peterson, Mayra Perez-Fajardo, Cole Peterson, Adina L. Santana, Conrad Kabus, Ryan Ardoin, Dmitriy Smolensky

**Affiliations:** 1 Grain Quality and Structure Research Unit, Center for Grain and Animal Health Research, Agricultural Research Service, U.S. Department of Agriculture, Manhattan, KS 66502, USA; mayra.perez-fajardo@usda.gov; 2 College of Business Administration, Kansas State University, 919 Mid-Campus Drive North, Manhattan, KS 66506, USA; 3 Department of Grain and Food Science, Kansas State University, 1301 Mid-Campus Drive, Manhattan, KS 66506, USA; adina@ksu.edu (A.L.S.); conradkabus@ksu.edu (C.K.); 4 Food Processing and Sensory Quality Research Unit, Southern Regional Research Center, Agricultural Research Service, U.S. Department of Agriculture, New Orleans, LA 70124, USA; ryan.ardoin@usda.gov

**Keywords:** sorghum, sorghum leaves, phytochemicals, antioxidants, 3-deoxyanthocyanidins, functional food

## Abstract

Rising rates of obesity and diabetes have increased demand for sugar-free products, while consumers also seek natural colorants and foods offering functional health benefits. Sorghum leaves, an underutilized part of the plant, contain health-promoting polyphenolic compounds with bioactive and coloring properties. Incorporating sorghum leaf-derived polyphenols into foods may meet both producer and consumer interest in natural colorants and value-added ingredients. This study aimed to incorporate sorghum leaf polyphenol extracts into sugar-free gelatin candies. Four formulations were produced: artificial-low color, natural-low color, artificial-high color, and natural-high color candies. Instrumental texture was evaluated using firmness and springiness measurements. Water activity was recorded using a water activity meter. Color was assessed with a colorimeter. Total phenolic content (TPC) was measured using the Folin–Ciocalteu assay. Consumers’ (N = 105) acceptance of sensory properties was evaluated using 9-point hedonic scales, and purchase intent was measured before and after colorant source and sweetener informational messaging. Natural colorant formulations had significantly higher water activity than artificially colored candies. Instrumental firmness and springiness differed significantly (*p* < 0.05) across formulations. However, these differences did not significantly (*p* > 0.05) impact consumer texture preference. TPC increased with higher levels of sorghum leaf extract but did not increase bitterness ratings. After receiving educational information about the colorant sources, purchase intent for the naturally colored formulations significantly (*p* < 0.05) increased, exceeding that of artificially colored candies. These findings demonstrate that sorghum leaf polyphenols can be successfully incorporated into sugar-free gummy candies and are well accepted by consumers as a natural colorant. This work supports sorghum as a value-added food ingredient, creating new market opportunities for US sorghum farmers.

## 1. Introduction

Gummy candies are a popular, familiar candy product for consumers of all ages. The United States (US) gummy candy market is a multi-billion-dollar industry estimated to grow from $3.67 billion in 2024 to over $10.97 billion by 2032 [1]. Gummy candies have also become an increasingly preferred carrier for delivering nutrients and bioactive compounds [2]. Their popularity is driven by their characteristic chewy texture, diverse flavors, color variety, and broad consumer appeal. Conventional gummy formulations typically incorporate gelatin as the primary gelling agent, along with corn syrup (to prevent crystallization), sucrose (for sweetness), flavorings, and food colorants. Despite their widespread use, these products are generally high in sugar and offer minimal nutritional value, limiting their suitability for consumers managing chronic conditions, such as obesity and diabetes.

The occurrence of obesity in the US has increased over the years, affecting more than 40% of adults as of 2023 [3]. To address this, food product developers have explored the use of alternative sweeteners, such as stevia to reduce sugar content without compromising sensory acceptability. A recent study examining the effect of stevia as a sucrose replacer found that gummy candies formulated with just 2.59% stevia was the optimal level of sweetness when assessed by 30 panelists using the 7-point hedonic scale [4].

In addition to nutritional concerns, consumer preferences have shifted toward natural colorants and clean-label ingredients [5]. Amid this trend, the US Food and Drug Administration (FDA) announced measures to phase out synthetic (artificial) food dyes in favor of new natural colorants [6]. Plant-derived extracts have increasingly been incorporated into confectionery products to replace artificial dyes while limiting adverse sensorial and functional attributes [7]. Although these advancements have expanded opportunities for functional confectionery, there remains a need for natural pigments that are not only stable and visually appealing but also capable of contributing bioactive properties.

In recent years, consumer demand has shifted to products that offer additional health benefits beyond basic nutrition. This shift has significantly influenced the development of functional foods that incorporate bioactive compounds into everyday products. Low-sugar gummy candies are an attractive vehicle for bioactive compounds as they offer a sweet taste that could help mask the undesirable flavor profiles of some functional ingredients, such as polyphenolic compounds from sorghum [8]. This is crucial for demographics that would otherwise refuse to consume bioactive compounds in tablet or capsule form [2] or directly from their native food source.

Sorghum (*Sorghum bicolor* (L.) Moench) represents a promising, yet underutilized, ingredient for use in such functional applications. Although the US is the world’s largest producer of sorghum, less than 2% of domestic production enters the food market; instead, most sorghum is directed toward low-margin channels such as ethanol production and animal feed. Sorghum’s agronomic advantages, including drought tolerance, high biomass yield, and low input requirements, positions it well for expanded use in higher-value food markets. Increasing demand for sorghum-based food products has the potential to enhance economic opportunities for US growers by elevating the crop from a commodity grain to a value-added ingredient source.

Specialty pigmented sorghum lines contain polyphenols and tannins with many reported health benefits including anticancer activity, antidiabetic, and anti-inflammation [9,10,11,12]. Additionally, the stalks and leaves of sorghum also contain health-promoting polyphenols [13,14,15]. Similar bioactive-rich plant extracts from fruits and teas have been successfully incorporated into gummy formulations; however, no studies to date have examined the integration of sorghum-derived polyphenols into low-calorie, gelatin-based gummy confections. Understanding how sorghum bioactives influence gummy texture, color stability, and consumer acceptance is essential for evaluating their feasibility as natural colorants and functional ingredients.

For this reason, the proposed work functions as an essential proof-of-concept study designed to assess the incorporation of sorghum leaf extracts into gummy matrices. This study tests the hypothesis that sorghum leaf bioactives may serve as a natural colorant alternative to synthetic petroleum-based dyes with minimal influence on physical and sensory attributes in low-sugar gummy candies. Findings from this study will provide foundational data necessary to support future research into the nutritional and health-related benefits of sorghum bioactives in functional foods. Furthermore, successful development of sorghum-based candies could simultaneously meet consumer demand for naturally colored, functional confectionery products and create new value-added markets for US sorghum farmers.

## 2. Materials and Methods

### 2.1. Materials

A red leaf sorghum extract was purchased from RedLeaf Biologics (Lexington, KY, USA). The food-grade ingredients for the gelatin candies (all food grade) were allulose (Rx Sugar, Atlanta, GA, USA), water, stevia (Kroger Co., Cincinnati, OH, USA), gelatin (Knox Gelatine, Chicago, IL, USA), citric acid (Mrs. Wages, Moorhead, MN, USA), strawberry and raspberry extracts (McCormick and Company, Hunt Valley, MD, USA), coconut oil (Good & Gather, Minneapolis, MN, USA), food coloring (Red 40, Red 3, Yellow 5, Yellow 6, Blue 1, Blue 2, and Green), and corn starch (Kroger Co., Cincinnati, OH, USA). All reagents for analysis were analytical grade. Gallic acid and sodium bicarbonate were purchased from Thermo Fisher Scientific (Waltham, MA, USA). Folin–Ciocalteu (FC) reagent was purchased from Sigma-Aldrich (St. Louis, MO, USA).

### 2.2. Gelatin Candy Preparation

In this study, gummy candy formulations underwent preliminary optimization to identify a workable base formulation with acceptable taste, mouthfeel, appearance, and flavor. These steps were conducted solely to ensure the feasibility of incorporating sorghum leaf extract into a gummy matrix and to evaluate initial consumer acceptance. The formulation was intentionally developed to match the appearance of commercially available gummies colored with artificial dyes so that the study could assess whether sorghum extract could function as a potential replacement. As such, the products produced here represent a proof-of-concept rather than a finalized, consumer-ready formulation. Additional research will be required to refine processing parameters, evaluate food safety and storage stability, and determine appropriate formulation adjustments for long-term commercial viability. Formulation ingredient results are presented in Table 1. Four gummy candy formulations were created to test the application of sorghum leaf extracts at two separate concentrations in comparison to artificial colorants.

Gelatin gummy candies were formulated by dissolving stevia in pre-dyed water at 100 °C (±1 °C) for 8 min. After dissolving stevia, allulose was added and stirred until dissolved. Gelatin and citric acid were slowly dissolved and stirred over medium heat for 4.5 min in the allulose–water mix. Once fully dissolved, flavoring and oil were added before entire gummy candy mix was added to a squeeze bottle. The mix was left to stand for 20 min at room temperature before pouring into the molds. During pouring, inversion of the bottle was minimized to limit bubble formation in the gummy candy. After resting at room temperature for 20 min, candies were stored at 20 °C overnight. After refrigeration, candies were separated from molds and coated with corn starch to prevent sticking. Candies were then packaged for analysis. One manufacturing batch was produced, and all measurements were carried out using analytical replicates.

### 2.3. Water Activity

Water activity (a_w_) was measured using a PAWKIT portable water activity meter (Aqualab, Pullman, WA, USA). Three gummy candies were placed in the provided stainless-steel cup for each analysis to limit open space during testing. Results were reported as the mean ± standard deviation of 3 separate measurements.

### 2.4. Texture

Texture was measured using the stable microsystems TA.XT plus texture analyzer (Texture Technologies Corporation, Hamilton, PA, USA). Samples were analyzed for firmness and springiness using the pre-programmed “Gummy Confectionary” test with the following modifications: pre-test speed 1.0 mm/s, test speed 10.0 mm/s*, post-test speed 10.0 mm/s, strain 50%, time 1 s*, and trigger type Auto 5 g. Gummy candies were laid on their side for testing to reduce sample variability, and n = 20 gummy candies were selected randomly from each batch for analysis.

### 2.5. Color

Prior to processing, the colorants were matched to one another using a portable MiniScan EZ 4500L colorimeter from HunterLab (Reston, VA, USA). After processing, artificial gummy candy colorants visually changed. For this reason, color was analyzed again using a Konica Minolta CM-5 spectrophotometer (Osaka, Japan) and changed accordingly prior to pouring treatments into molds. The artificial color was altered post-processing to limit visual differences for subsequent sensory testing. Between the two types of colorings (artificial vs. natural), a delta E color difference calculation was performed according to Equation (1).

(1) $$∆E^{*}=\sqrt{{\left(L_{2}^{*}-L_{1}^{*}\right)}^{2}+{\left(a_{2}^{*}-a_{1}^{*}\right)}^{2}+{\left(b_{2}^{*}-b_{1}^{*}\right)}^{2}}$$

### 2.6. Total Phenolic Content

The Folin–Ciocalteu method was used according to Herald, Gadgil [16], with modification. All standards and samples were performed in deionized water. A standard curve of gallic acid ranging from 0 to 800 µg/mL (R^2^ = 0.99) was used, with absorbance of samples and standards read at 715 nm. Results are expressed as milligram gallic acid equivalents per gram of sample (mg GAE/g sample) as the mean ± standard deviation.

### 2.7. Sensory Evaluation

One hundred and five adult consumers (N = 105) were recruited to participate in this study. Participants included university students, faculty, staff, and members of the local community. Participation was incentivized with a coupon for free ice cream. Protocols for ethical human subjects’ research were reviewed and approved by the LSU AgCenter Institutional Review Board (IRBAG-22-0017), and all consumers provided informed consent prior to participation.

Testing was conducted in partitioned sensory evaluation booths at the Louisiana State University AgCenter Sensory Services Lab (Baton Rouge, LA, USA). Each consumer received all four gummy candy samples to evaluate one at a time. Samples were coded as follows: artificial-low (394), natural-low (532), artificial-high (840), and natural-high (167). Sample evaluation order was randomized and balanced across participants. Unsalted crackers and water were served for palate cleansing. Consumers first answered demographic questions (gender, age, race, ethnicity, and frequency of gummy candy consumption/purchase). For each sample, consumers rated color, aroma, texture, flavor, and their overall liking (OL) of the gummy candy on a words-only 9-point hedonic scale (from “dislike extremely” to “like extremely”). Sweetness and bitterness were rated on a 3-point just-about-right (JAR) scale (“not sweet enough,” “just about right,” or “too sweet”; “no bitterness” (considered the JAR category), “weak bitterness,” or “strong bitterness”). Purchase intent (PI) was rated (“yes” or “no” scale) three times, sequentially, for each sample: with no ingredient information (blind), with colorant information (CI), and with sweetener information (SI). For samples made using artificial colorants (artificial-low and artificial-high), the following CI was presented: “Gummy candy sample [XXX] was made with food colorings: Red 40, Red 3, Yellow 5, Yellow 6, Blue 1, Blue 2, and Green.” For samples made with natural colorant from sorghum leaves, the following CI was presented: “Gummy candy sample [XXX] was made with natural colorant from sorghum leaves which contain natural antioxidants like those found in berries, green tea, and red wine.” The same SI was presented for all samples: “This sample is sugar-free and made with no-calorie sweeteners.” Finally, consumers reported their familiarity with sorghum before participating in the trial (had NOT heard of nor eaten it, had heard of but NOT eaten it, or had heard of and eaten it).

### 2.8. Statistical Analysis

For water activity, texture, and TPC, one-way analysis of variance (ANOVA) with Tukey’s post hoc testing using GraphPad Prism, version 10.1.0 (GraphPad, San Diego, CA, USA) was used. To demonstrate differences in colorant source, concentration, and their interaction on water activity, texture, and color, a two-way analysis of variance (ANOVA) with Tukey’s post hoc testing was used, and the results are reported in Supplementary Table S5. Significant difference in mean values was determined at *p*-value < 0.05. Assumptions of normality were confirmed for all variables except artificial-low gummy candy a* values (Shapiro–Wilk tests). Heteroscedasticity was confirmed for all variables but firmness (Bartlett’s tests). Considering only minor violations of assumptions and equal sample sizes, parametric analyses were used with these instrumental data.

Due to marked departures from normality in sensory data, Friedman’s tests were used to investigate differences in consumers’ hedonic scores across the four samples (for color, aroma, texture, flavor, and OL, respectively). Wilcoxon signed-rank tests were used for post hoc pairwise comparisons with a Bonferroni correction to control family-wise error rate. Changes in positive purchase intent (PI = yes) frequencies upon each additional information level were analyzed by McNemar’s tests for marginal homogeneity. JAR data were subjected to penalty analysis to explore the impact of non-JAR responses for sweetness and bitterness on mean OL of samples.

## 3. Results

### 3.1. Gummy Candy Preparation

**Figure 1:**
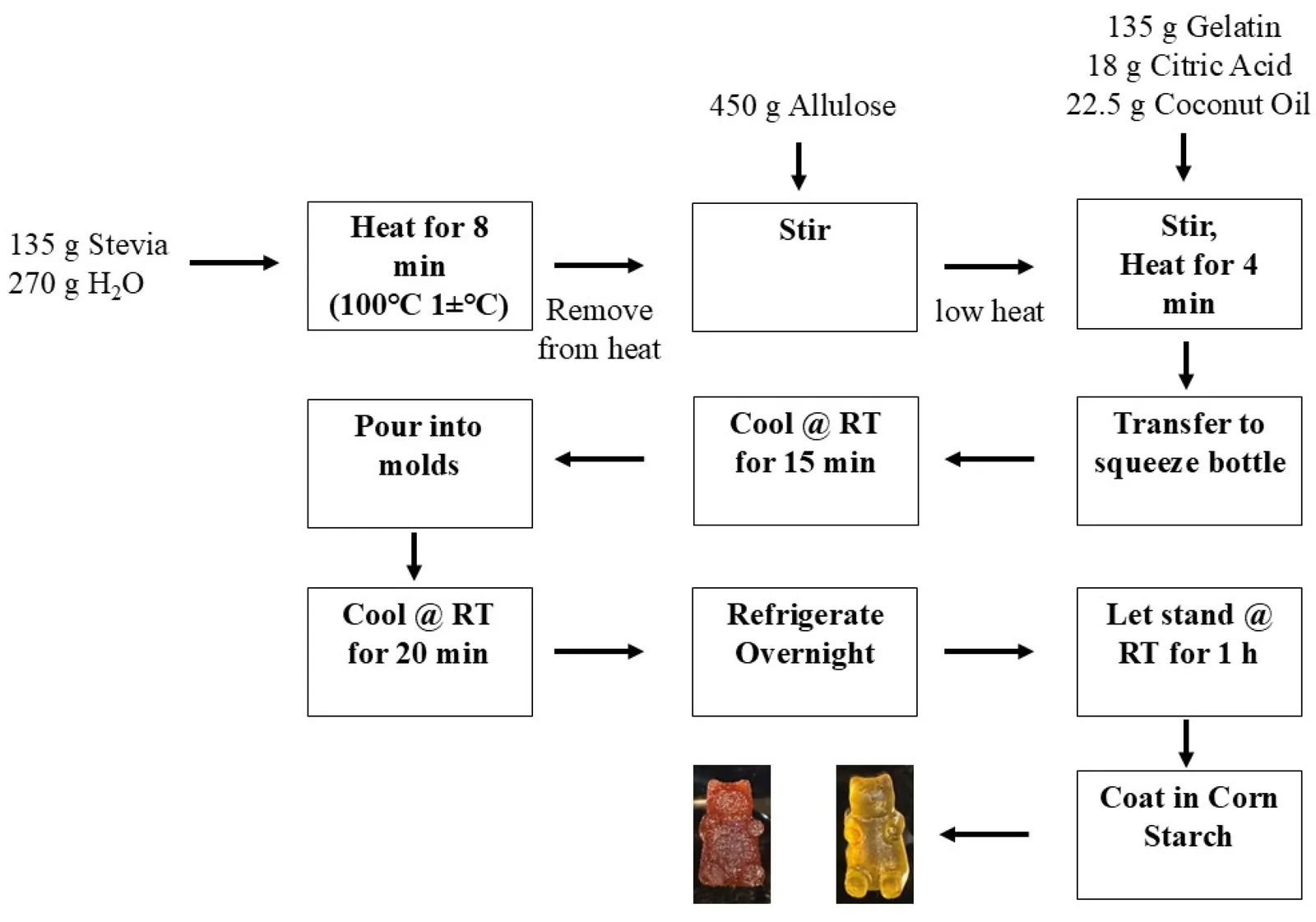
Process flow diagram.

To assess the ability of sorghum leaf extract to be used as a heat stable, value-added, bioactive colorant, multiple preliminary formulations were conducted. This research aimed to choose the best formulation for gummy candies with final sensory applications in mind. That is, parameters like appearance, mouthfeel, flavor, size, and safety were considered during formulation to yield the best final product for consumer acceptability. It was further decided that only sugar-free formulations be investigated. The process flow diagram for gummy candy formulation is displayed in Figure 1. The overall gummy candy composition of the selected formulas is displayed in Table 1.

### 3.2. Physical Characterization

**Figure 2:**
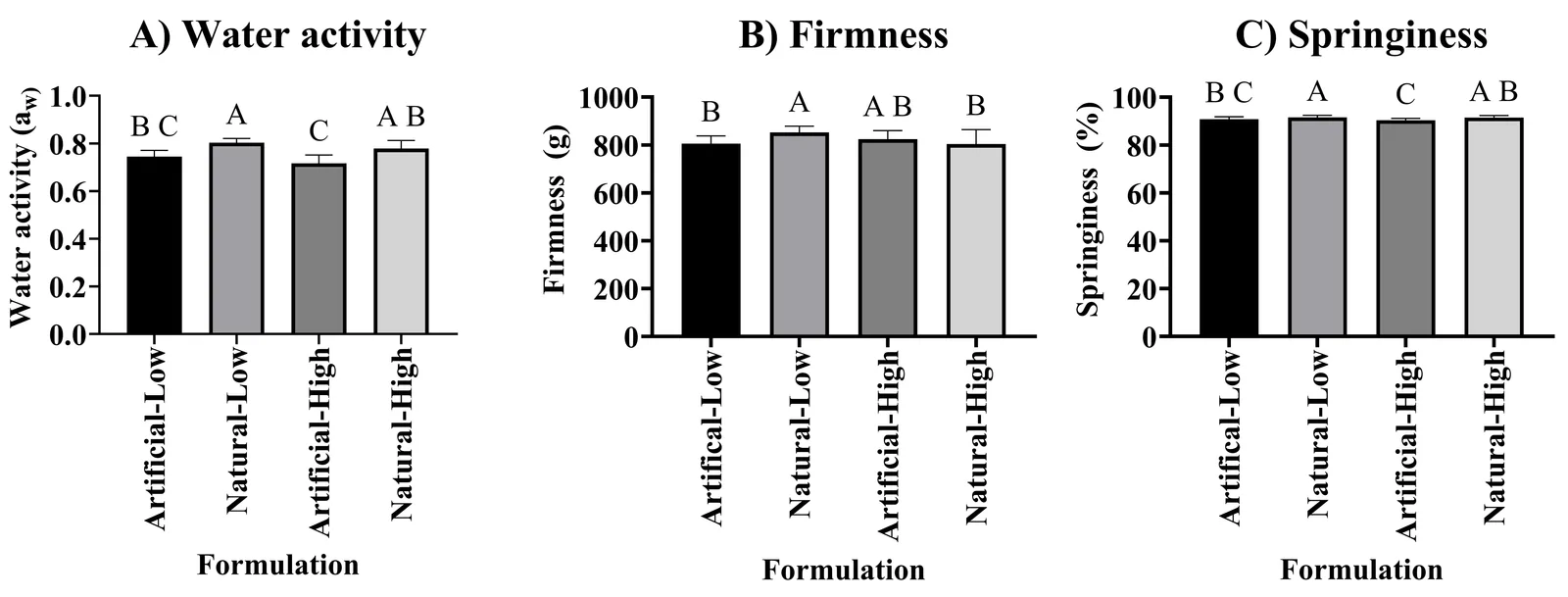
Physical characterization of gummy candy formulations using one-way ANOVA; (**A**) water activity, (**B**) firmness, and (**C**) springiness. Values represent mean ± standard deviation. Samples with different letters are statistically significant (*p* < 0.05).

The water activity (a_w_) of samples ranged from 0.72 to 0.80 (Figure 2A) with significant overall effects of both colorant source and concentration (Supplementary Table S5). The artificial-low dose was significantly lower (*p* < 0.05) than the natural-low color formulation. This trend was repeated when comparing the artificial-high color and natural-high color formulations. When comparing low and high colorant incorporations, there were no significant differences within colorant type.

Regarding instrumental firmness, statistical differences were attributed to the interaction between factors (colorant source and concentration, Supplementary Table S5, *p*-value < 0.05) rather than main effects. The natural-low formulation was significantly firmer than the artificial-low color and natural-high color formulation (Figure 2B). In terms of firmness, the natural-low and artificial-low candies were significantly different from one another, whereas both high dose formulations were not significantly different from one another. The natural low-dose gummies were significantly firmer than the natural high-dose gummies.

For springiness, significant differences between samples were observed based on colorant source (Supplementary Table S5). The natural-low color formulation (91.54%) was significantly higher than the artificial-low (Figure 2C). Likewise, the natural-high color formulation (91.49%) was significantly higher than the artificial counterpart (90.4%). There were no statistical differences between the natural-low and natural-high formulation or between the artificial-low and artificial-high color formulations when compared to each other.

### 3.3. Color Analysis

The color (Table 2) observed in the different formulations was primarily associated with the concentration of colorant (low vs. high; Supplementary Table S5, *p* < 0.0001). For example, the low colorant samples were higher in L* values (lightness) than the high colorant formulations. Likewise, the high colorant formulations have higher a* and b* (more red and yellow, respectively) values in comparison to their low colorant counterparts. Colorant source (artificial vs. natural) also affected instrumental color measurements (with a significant interaction observed for a* values (Supplementary Table S5, *p* < 0.0001).

Calculating Delta E (ΔE) represents the Euclidean distance from two coordinates in the three-dimensional color space. It is used as a standard calculation to determine human visual assessments of differences between two colors. A Delta E value of 2 or less is generally considered not noticeable to consumers; any color difference at that level can only be seen under close inspection [17]. The delta E value between the low-dose samples was 1.23, and the delta E value between the high-dose samples was 1.39.

### 3.4. Total Phenolic Content

**Figure 3:**
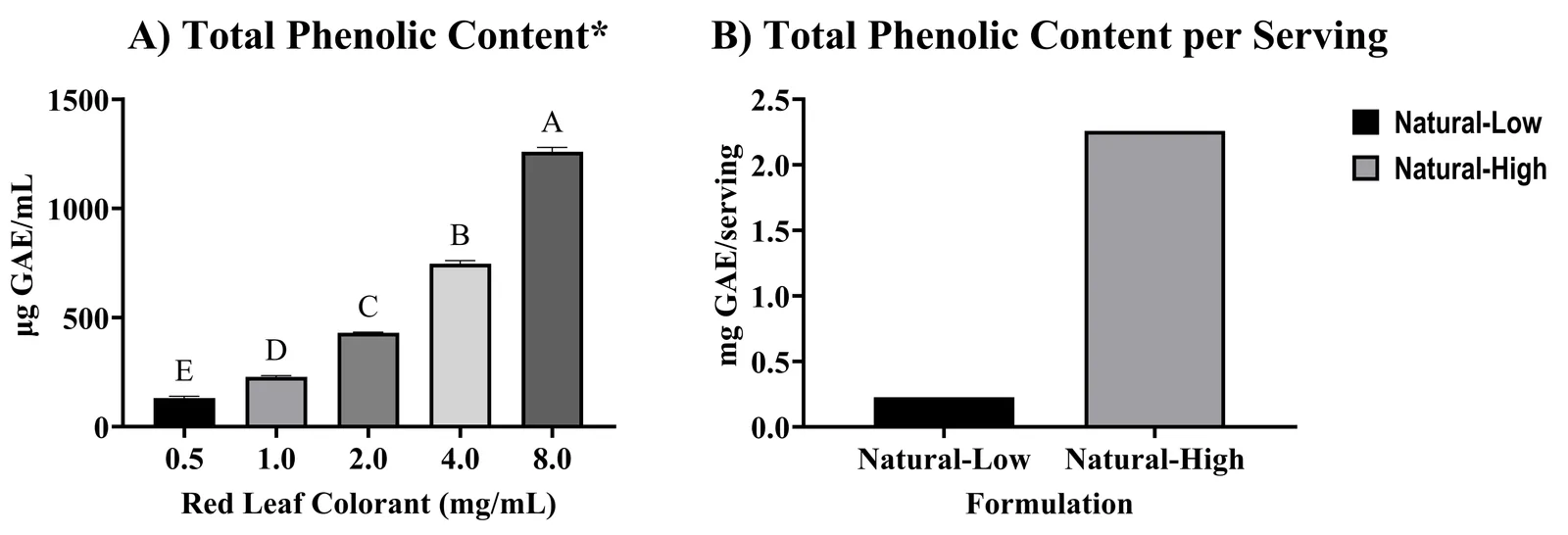
The total phenolic content of various (**A**) red leaf sorghum extract concentrations used during preliminary studies and (**B**) the total phenolic content per serving of gummy candies (30 g) at each dosage selected for analysis. * Samples with different letters are statistically significant (*p* < 0.05) and represent the mean ± the standard deviation of three separate analyses.

During primary analysis, the total phenolic content (TPC) of the red leaf extract was examined at different concentrations. This was done to ensure the red leaf extract could adequately dissolve prior to mixing with gummy candy ingredients. As expected, the TPC of the extract increased with red leaf concentration. For example, the highest dose, 8.0 mg/mL red leaf extract, had the highest TPC (1259 µg GAE/mL) when compared to all other concentrations (Figure 3A). Additionally, doubling the concentration of extract increased the TPC by an average of 42.5% each time. This indicates that the extract is soluble in aqueous solutions.

From the preliminary TPC analysis, a natural low dose (0.1 mg/mL extract) and a high dose (1.0 mg/mL) were selected for formulation. These extract concentrations were intentionally kept low to visually match the appearance of commercially available gummy candies colored with artificial dyes. Higher levels would have produced considerably darker gummies, preventing a comparable evaluation of natural versus artificial colorants in consumer testing. Higher extract doses will be required in future studies aimed at evaluating antioxidant activity, bioavailability, or functional properties. After selecting the dosage levels, the TPC per 30 g serving size was calculated. The low-dose concentration had 0.22 mg GAE per serving, while the high dose was 2.26 mg GAE per 30 g serving size (Figure 3B).

### 3.5. Sensory Analysis

The sociographic data of sensory participants are displayed in Table 3. Overall, participants identified as White (82%), Other (8%), Black/African American (6%), or Asian (6%). Additionally, 12% of participants further identified as Hispanic or Latino ethnicity. After reporting their race and ethnicity, participants were asked how often they regularly consumed or purchased gummy candies. Most participants (38%) reported consuming or purchasing gummy candies at least a couple times a year. To test the participants’ base familiarity with sorghum, they were asked about their general familiarity with sorghum. Most participants reported that they had heard of sorghum but had not personally eaten it (48%), while 34% of participants reported that they had neither heard of nor eaten sorghum prior to their participation.

Across the four formulations, significant differences were found for consumers’ liking of color and overall liking (OL) of gummy candies based on Friedman’s tests (α = 0.05). For the artificial-high and natural-high colored candies, participants rated them similarly in aroma, texture, flavor, and OL. The natural-high color candies were rated significantly higher in color liking than their artificially colored counterparts. In terms of overall liking (OL), the natural-high colored gummy candy was rated directionally highest and significantly more liked than the natural-low colored candy.

Participants rated the artificial-low and natural-low colored gummy candies similarly in terms of color, aroma, texture, and flavor (Table 4). Interestingly, when asked to examine the candies’ overall quality, participants rated the artificial-low color gummy candy directionally higher in OL than the natural-low colored candy. However, this difference would only be considered statistically significant upon comparison within the low-concentration group singularly (pairwise Wilcoxon *p*-value < 0.05), rather than across samples. The same would be true if directly comparing texture within the high-concentration group alone, where the artificial-high colored candy received directionally higher texture scores.

**Figure 4:**
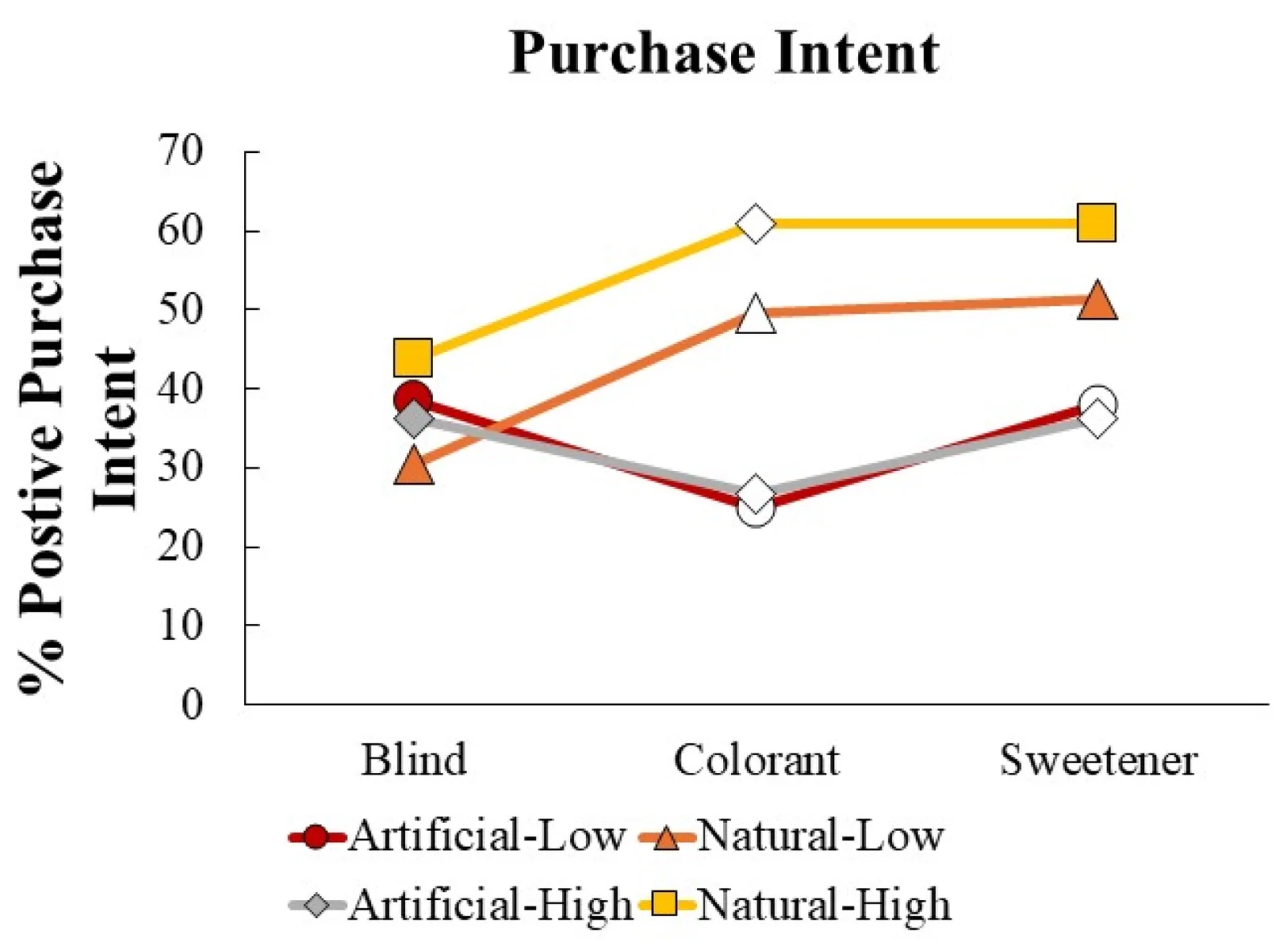
Purchase intent for gummy candies under blind tasting (no ingredient information), with colorant information 1, 2, and with sweetener information 3. ^1^ Artificial colorant message: “Gummy bear sample [###] was made with food colorings: Red 40, Red 3, Yellow 5, Yellow 6, Blue 1, Blue 2, and Green. ^2^ Natural colorant message: “Gummy bear sample [###] was made with natural colorant from sorghum leaves which contain natural antioxidants like those found in berries, green tea, and red wine.” ^3^ Sweetener message [all samples]: This sample is sugar-free and made with no-calorie sweeteners. ◊ Indicates a significant shift in purchase from the previous condition, based on McNemar’s test (α = 0.05).

Positive purchase intent for natural-low colorant candies was 30.5% and 43.8% for natural-high colorant candies prior to the educational messaging (Table 4). Following the initial purchase intent question, colorant and sweetener information was given for each sample sequentially. For the naturally colored candies, the positive purchase intent significantly increased after receiving the colorant message to 49.5% for the low dose and 61% for the high dose. The purchase intent of natural candies did not significantly change after the low-calorie sweetener message. After receiving the colorant messaging, the positive purchase intent for both artificially colored gummy candies significantly decreased to around 25% (Figure 4). After the sweetener message, the purchase intent significantly increased to 38.1 (low dose) and 36.2% (high dose). Overall, the purchase intent of naturally colored candies was significantly higher after educational messaging than the artificial candies.

**Figure 5:**
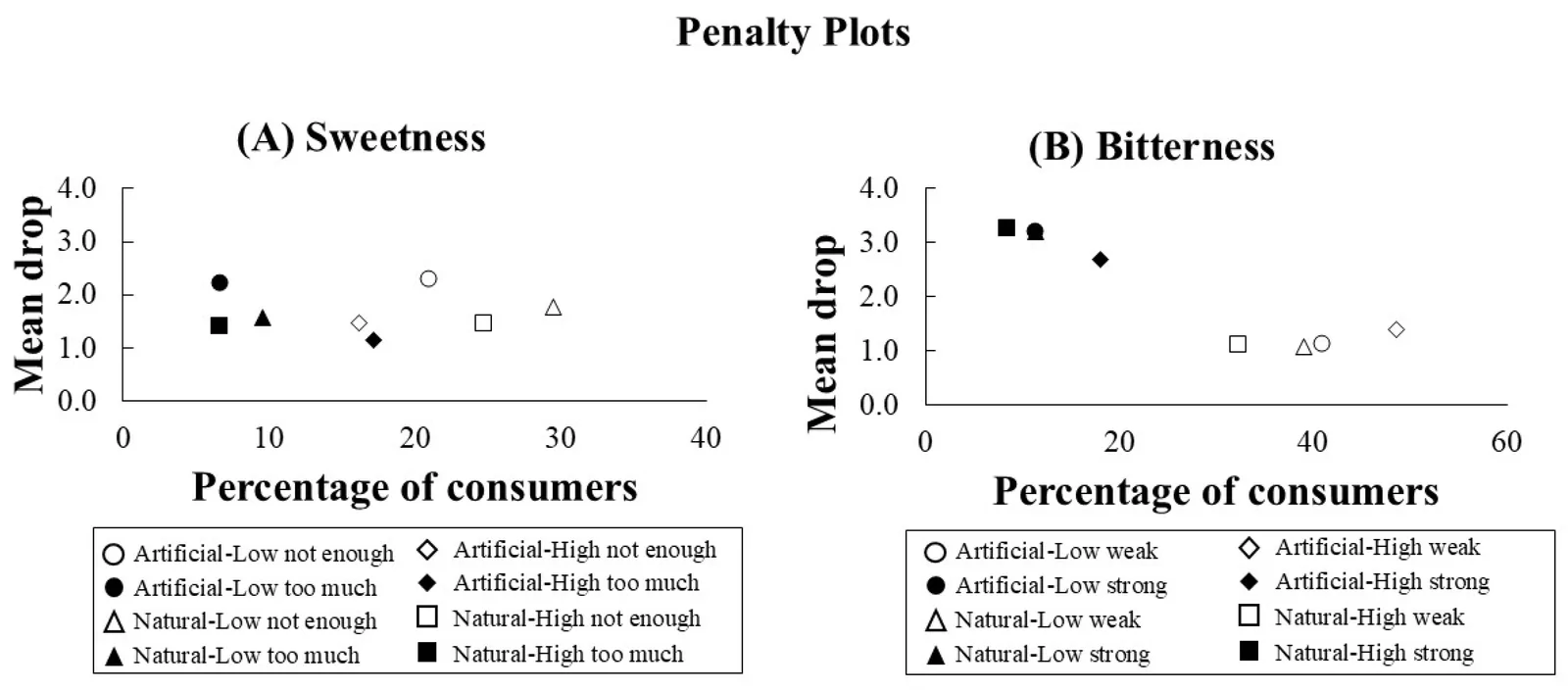
Penalty plots for just-about-right (JAR) (**A**) sweetness and (**B**) bitterness data. *X*-axis represents percentage of non-JAR ratings (from N = 105 consumers). *Y*-axis represents the average difference in overall liking (OL; 9-point scale) for non-JAR responses compared to JAR responses for each sample.

Based on a heuristic from Rothman [18], non-ideal JAR responses for sensory attributes may be considered concerning when selected by at least 20% of respondents with an average drop in acceptability (penalty) of at least 1.5 units. Artificial-low color, natural-high color, and natural-low color samples were rated as not sweet enough by between 21% and 30% of consumers, resulting in average OL penalties up to 2.3 units on the 9-point hedonic scale (Figure 5). For all samples, less than 20% of consumers reported strong bitterness. However, weak bitterness was found by between 32% and 49% of consumers across formulations; no penalties exceeded 1.5 units. The naturally colored formulations received a higher proportion of “no bitterness” responses and lower proportions of both weak and strong bitterness ratings compared with the artificially colored formulations.

## 4. Discussion

Physical characteristics are not only important to consumers but are also necessary to maintain product quality and safety from a food safety perspective. For example, water activity is an important factor for evaluating microbial stability, texture, and water migration during storage [19]. In general, the reported acceptable water activity range for gummy confectionary candies to limit microbial growth is between 0.45 and 0.75 [20]. The naturally colored gummy formulations were slightly higher than this range, 0.8 for natural-low and 0.77 for natural-high. Although elevated relative to commonly recommended targets for gummy products, both values remained below the FDA’s critical limit of 0.85 for foods not requiring refrigeration [21,22]. This implies that the formulations were still within legally acceptable bounds for shelf-stable products. It should be noted, however, that water activity levels above 0.75 can increase the risk of spoilage by osmophilic yeasts and molds and may contribute to undesirable quality changes during storage, such as weeping or texture softening. Additionally, the higher water activity observed in natural formulations may be partially attributable to the hydrophilic characteristics of the sorghum polyphenol extract. The increase in water activity after the inclusion of natural sorghum polyphenol extracts is in line with similar studies, who also reported an increase in water activity after incorporating pomegranate juice into jelly candies [23]. It should also be noted that alternative sweeteners, such as allulose, exhibit different water-binding properties compared with traditional sucrose–corn syrup systems [24,25]. Given that the formulations in this study contained allulose, this ingredient may have contributed to the elevated water activity observed across all treatments, regardless of colorant type. Because an optimal “sweet spot” for gummy candy stability is typically closer to a water activity of 0.60, future studies incorporating alternative sweeteners should evaluate both formulation and processing modifications to reduce finished water activity. For example, increasing cooking time may promote additional water evaporation, thereby lowering the final water activity, thus improving microbial stability, shelf life, and overall storage performance.

In the creation of a novel food product, sensory and texture analysis is crucial, as it is heavily linked to the sensorial experience, mouthfeel, and overall consumer acceptability of a product. The firmness, which is defined as the peak force needed to deform or compress a sample, showed no clear correlations between the use of natural or artificial colorants. Springiness, which is defined as the rate at which deformed materials return to their original state after the force is removed, showed different trends. For example, naturally colored candies showed a clear correlation for increased springiness when compared to their artificial counterpart. The results differed from previous studies using orange peel extracts in gummy candies, where springiness was not affected [26]. Likewise, in another study examining the textural properties of jelly candies with purple basil leaf extracts, springiness was also not affected [27]. The differences in reported findings could be explained by several aspects such as the water content, type of gelling agent, sugar type, and bioactive compound source [19]. Future high-polyphenol formulations may need higher gelatin levels to reduce textural differences, as phenolic compounds can affect gelatin’s water-binding ability [28].

Given that color is a strongly appealing characteristic of gummy candies, it is important for product evaluation. Based on the CIELAB color results, the calculated delta E values between colorants were both below 2 which is believed to be optically indistinguishable [17]. Based on the hedonic scores, however, there were no differences at the low dose but a significantly higher preference for the color of the naturally colored high-dose candy in comparison to its artificial counterpart. This is in line with other findings that reported an increase in color after adding pomegranate extracts to jelly candies [29]. A possible explanation for the differences between the instrumental color measurements and consumer preferences is the timing of when each assessment was made. The candies were instrumentally tested for color differences immediately after processing, before they had fully set. In contrast, consumers visually evaluated the candies after the setting period. This is in line with Vojvodić Cebin, Bunić [30], who observed color changes over time during storage after using mountain germander extracts to naturally color gummy candies. Additionally, the natural colorant contains antioxidative compounds that can inhibit oxidative browning by scavenging free radicals, thus resulting in a brighter color after setting [31]. However, it should be noted that color stability was not evaluated in this study, and any explanations for the observed differences remain speculative until future work examines color changes over time. Given that the main difference between formulations was the presence or lack of natural colorants, this hypothesis is further supported by the fact that gummy candies were formulated with stevia, which contain colorless stevia glycosides that do not participate in caramelization or Maillard reactions [32].

The phenolic content of the extracts increased proportionally with dose, demonstrating clear water solubility and dose-dependent behavior. It is also well reported that increasing the total phenolic content will increase the antioxidant activity of a given food matrix [33]. Given the complex nature of the gummy candies, polyphenols could not be extracted in high enough concentrations for accurate TPC or antioxidant measurements. Therefore, future studies should focus on higher phenolic inclusions to examine the effect of increased extract amount on TPC and AA retention post-processing. Future studies should also examine the unique nature of protein–polyphenol interactions being both reversible and irreversible [34]. Furthermore, sugars and syrups are also known to contain polyphenols [35] and affect polyphenol extractability [36] and should also be considered for future applications.

Sensory analysis is an essential tool for evaluating formulation success and predicting consumer acceptance of new functional foods, especially given the potential tradeoff between healthfulness and enjoyment [37]. To mitigate this perceived tradeoff, optimal “healthier” formulations should match the reference product in sensory quality [38]. For natural-high polyphenol gummy candies, this was achieved for aroma, texture, flavor, and OL, while mean color scores were improved from 5.7 (artificial-high color) to 6.3 (natural-high color) (Table 4). For natural-low colorant samples, the use of sorghum leaf extract did not significantly impact liking scores compared to artificial-low color candies in the blind tasting condition. As bitterness associated with polyphenolics can cause consumers to avoid potentially health-promoting foods [39], a key finding from this study was that consumers perceived candies containing sorghum leaf extract as less bitter than artificially colored candies based on JAR data (Figure 5).

Sensory results indicated that increasing sorghum leaf extract dosage did not affect aroma or flavor, suggesting that the extracts themselves do not negatively influence these attributes and may therefore serve as viable natural colorants. This contrasts with studies that reported significant reductions in consumer acceptability when higher amounts of watermelon extract powder were incorporated into agar-based gelatin candies [40]. These differing outcomes may be due to the presence of agar, as certain hydrocolloids have been shown to influence texture and modify the release of aroma and flavor compounds [41]. Taken together, this suggests that overall gummy hardness and water activity are more influential determinants of consumer preferences for texture and aroma than extract dosage alone. This interpretation is further supported by studies using spirulina and acai mixtures in gelatin candies, which similarly reported no significant differences in consumer acceptability between formulations [42]. As in our study, texture acceptance did not differ across treatments, indicating that characteristics of the gummy matrix itself were the primary factors influencing sensory outcomes rather than the presence of sorghum leaf extract.

While consumers’ hedonic ratings displayed minimal differences among samples, their purchase intent revealed clear effects of extrinsic information on gummy acceptability. Significant increases in sorghum-based gummy purchase intent after CI (Table 4; Figure 4) align with reports of nutritional knowledge and health benefits as the most important drivers of functional food acceptance [37]. It should be noted that CI for samples containing sorghum leaf extract provided natural antioxidant messaging, which may have influenced consumers beyond ingredient naming alone. In contrast, perceived unnaturalness associated with the artificially colored candies’ CI may have caused concerns about product safety [37]. Specifically, synthetic dyes Red 40, Yellow 5, Yellow 6, Blue 1, and Blue 2 were also mentioned in the FDA’s phase-out plans, which may reflect consumers’ concerns [6]. Compared to blind tasting alone, data from the informed conditions (after CI and SI) were more representative of actual purchase scenarios where consumers’ choices are influenced by contextual information [43]. Though “sugar-free” SI seemed to compensate for the drop in artificial gummy purchase intent, sorghum leaf-colored candies showed the most potential with consumers (Figure 4), indicating clear consumer demand for value-added foods derived from natural sources. Combining sensory quality with product information, consumers were more accepting of sorghum leaf extract as a colorant source than artificial dyes, despite only 20% reporting previous sorghum consumption.

This proof-of-concept study was designed to evaluate the feasibility of incorporating sorghum leaf polyphenol extracts into sugar-free gummy candies and to assess initial consumer acceptance. However, several limitations should be noted. First, although the extract itself was characterized for total phenolic content, the phenolics and antioxidant activity of the finished gummy candies could not be reliably measured due to challenges in extracting polyphenols from the complex gummy matrix at the intentionally low dosing levels used. Second, sorghum leaf incorporation was purposefully kept minimal to match the appearance of current artificial colorants for sensory testing; future studies should explore higher inclusion levels to better understand both sensory impacts and polyphenol retention. Third, the color stability was not evaluated, and any explanation for differences between instrumental and sensory colors assessments remain speculative until further work examines color retention. Fourth, the water activity of the naturally colored formulations exceeded the recommended range for microbial stability, indicating the need for additional shelf-life studies as well as formulation adjustments aimed at reducing moisture content during processing. Finally, only one manufacturing batch was produced for this study; therefore, future research should incorporate multiple batches to validate the consistency and reproducibility of the observed trends.

## 5. Conclusions

Overall, this study demonstrates that sorghum leaf polyphenol extracts can be successfully incorporated into gummy candies without compromising key sensory attributes. These findings support a promising opportunity for the sorghum and food industries, particularly as a clean-label alternative to synthetic and artificial dyes. Although the water activity in the natural formulations slightly exceeded the ideal range for microbial stability, this challenge can be addressed through future adjustments to the matrix. In addition, the extract itself did not negatively influence color, aroma, or flavor acceptance. Because texture differences stemmed from matrix properties, not extract dosage, future studies should focus on improving the formulation rather than lowering natural colorant levels. Importantly, despite instrumental color differences being minimal, consumers showed a clear preference for the appearance of candies with higher natural extract levels. Sensory acceptance and purchase intent results show that sorghum-derived natural colorants are strong replacements for artificial dyes and align with consumer demand for clean-label, plant-based, value-added foods. These results indicate that sorghum leaf polyphenols can function as a natural, consumer-accepted alternative to artificial food colorants. Future studies should incorporate a health-focused component and evaluate shelf life to more fully characterize the functional potential and stability of sorghum-colored gummy formulations.

## Figures and Tables

**Table 1:** Gummy candy composition.

	Artificial-Low	Natural-Low	Artificial-High	Natural-High
Ingredients	(g)	(g)	(g)	(g)
Allulose	450	450	450	450
Water	270	270	270	270
Gelatin	135	135	135	135
Stevia	135	135	135	135
Flavoring	90	90	90	90
Coconut Oil	22.5	22.5	22.5	22.5
Citric Acid	18	18	18	18
Colorant Type	Artificial *	Sorghum Leaf Extract	Artificial *	Sorghum Leaf Extract
Colorant Amount	9.48	0.027	39.21	0.27
Total	1129.98	1120.53	1159.71	1120.77

* Artificial colorants were a blend of petroleum-based synthetic dyes Red 40, Red 3, Yellow 5, Yellow 6, Blue 1, Blue 2, and Green.

**Table 2:** Instrumental color of gummy candies ^1^.

	L*	a*	b*	Δ-E ^#^
Artificial-low	19.20 ± 0.39 ^a^	1.10 ± 0.07 ^c^	7.58 ± 0.14 ^b^	1.23
Natural-low	19.78 ± 1.03 ^a^	0.19 ± 0.09 ^d^	8.19 ± 0.63 ^b^	
Artificial-high	13.44 ± 0.37 ^b^	6.36 ± 0.23 ^b^	13.58 ± 0.40 ^a^	1.39
Natural-high	14.62 ± 0.14 ^b^	6.86 ± 0.15 ^a^	14.13 ± 0.14 ^a^	

^1^ Expressed as mean L* a* and b* values ± standard deviation from three replicates. ^#^ Delta-E (Δ-E ^#^) color difference. ^a, b, c, d^ Values within a column followed by different letters were statistically different (ANOVA with post hoc Tukey test *p* < 0.05).

**Table 3:** Sociographic percentages and (frequencies) based on N = 105 consumer responses.

Gender	64% (67) Female, 36% (38) Male
Race ^1^	Asian 6% (6), Black or African American 6% (6), Other 8% (9), White 82% (86)
Ethnicity ^1^	Hispanic or Latino 12% (13), 88% (92) not Hispanic or Latino
Gummy consumption/purchase	Weekly 14% (15), monthly 33% (35), at least a couple times per year 38% (40), once a year or less 13% (14), never 1% (1)
Familiarity with sorghum	Had not heard and had not eaten 32% (34) Had heard but had not eaten 48% (50) Had heard and eaten 20% (21)

^1^ Race and ethnicity categories from US Census Bureau (2022) were used. Respondents could select more than one racial category. The US Census Bureau has since (2024) combined race and ethnicity into one question.

**Table 4:** Consumers’ hedonic scores ^1^ and purchase intent ^2^ for gummy candies.

	Artificial-Low (394)	Natural-Low (532)	Artificial-High (840)	Natural-High (167)
Color	6.1 ± 1.6 ^ab^	6.0 ± 1.6 ^ab^	5.7 ± 1.7 ^b^	6.3 ± 1.5 ^a^
Aroma	5.4 ± 1. 8	5.4 ± 1.7	5.5 ± 1.8	5.6 ± 1.6
Texture	4.9 ± 2.1	4.9 ± 2.0	5.3 ± 1.9	5.0 ± 1.9
Flavor	5.5 ± 2.1	5.5 ± 1.9	5.4 ± 2.0	5.7 ± 1.9
Overall	5.3 ± 2.1 ^ab^	4.9 ± 1.9 ^b^	5.2 ± 1.9 ^ab^	5.5 ± 1.8 ^a^
PI blind	38.5	30.5	36.2	43.8
PI with color info	24.8 *	49.5 *	26.7 *	61.0 *
PI with sweetener info	38.1 **	51.4	36.2 **	61.0

^1^ Mean value ± standard deviation from 9-point hedonic scale, based on 105 consumer responses. ^2^ Purchase intent (PI) expressed as the percentage of “yes” responses from 105 consumers. ^a,b^ Scores followed by different letters were significantly different between candies, based on Friedman’s tests with post hoc Wilcoxon tests (α = 0.05). Reported *p*-values for each attribute are reported in Supplementary Table S1. *^,^ ** A significant shift in PI percentage was observed upon delivery of new information, based on McNemar’s test (α = 0.05).

## Data Availability

The original contributions presented in this study are included in the article/Supplementary Materials. Further inquiries can be directed to the corresponding authors.

## Supplementary Materials

The following supporting information can be downloaded at: https://www.mdpi.com/article/10.3390/foods15173037/s1, Table S1: Friedman’s test results; Table S2: Texture and water activity data; Table S3: Color data; Table S4: Total phenolic content data; Table S5: Two-way analysis of variance results for effects of colorant source, colorant concentration, and their interaction on physical properties of gummy candies; File S1: Consumer responses.

## Abbreviations

The following abbreviations are used in this manuscript:

Anova	one-way analysis of variance
a_w_	water activity
CI	colorant information
FC	Folin–Ciocalteu
FDA	food and drug administration
GAE	gallic acid equivalents
IRB	institutional review board
JAR	just about right
OL	overall liking
PI	purchase intent
SI	sweetener information
TPC	total phenolic content
US	United States
ΔE	delta E, a standard metric used to quantify the differences between two colors
