# Supplementary material for: Development and Consumer Acceptance of Low-Sugar Gummy Candies Naturally Colored with Sorghum Leaf Polyphenols
Source: Foods. 2026 Aug 28;15(17):3037. doi: 10.3390/foods15173037 (PMC13565140; doi:10.3390/foods15173037)
Supplement: Supplementary file 1 [file foods-15-03037-s001.zip › Supplementary Tables S1-S5.pdf]

Supplementary Table S1. Friedman's test results[1]

**Attribute**	**P-value**
**Color**	0.0227
**Aroma**	0.5484
**Texture**	0.0361
**Flavor**	0.2465
**Overall liking**	0.0088

[1] Comparing hedonic scores (9-point scales) among 4 gummy candy samples based on 105 consumer responses.

Supplementary Table S2. Texture and water activity data

Treatment	Analysis	Recorded Measurement																				
	**Firmness (g)**	772.06	802.07	804.66	829.31	850.06	836.73	778.44	845.65	809.33	817.2	798.98	871.44	788.97	784.1	838.31	785.91	794.46	743.28	775.37	762.58	828.33
**Artificial-Low**	**Springiness (%)**	89.57	91.81	91.8	92.08	90.14	91.86	89.96	89.55	89.97	89.58	91.38	90.71	90.67	91.05	90.12	92.62	91.1	89.2	89.94	92.86	90.64
	**Water Activity**	0.82	0.81	0.79	0.83	0.79	0.8	0.78														
	**Firmness (g)**	871.91	859.33	783.56	829.8	881.42	874.24	864.45	879.19	840.37	819.78	811.9	878.22	853.03	853.09	867.06	819.7	851.77	882.31	847.86	856.7	869.98
**Natural-Low**	**Springiness (%)**	90.34	89.84	91.44	90.76	92.16	90.98	93.06	91.71	92.33	91.42	92.55	92.16	92.3	92.27	91.04	91.99	92.52	91.12	90.86	91.05	90.53
	**Water Activity**	0.84	0.8	0.79	0.75	0.77	0.75	0.75														
	**Firmness (g)**	824.81	829.09	807.16	907.32	827.75	816.78	836.39	829.25	894.57	808.62	843.82	838.57	781.95	820.25	775.49	822.64	779.94	753.74	811.14	834.48	859.7
**Artificial-High**	**Springiness (%)**	89.95	91.47	90.96	90.75	90.89	90.7	90.71	91.11	90.41	89.54	90.55	90.67	91.16	89.86	89.93	88.71	89.92	90.17	91.14	90.72	89.01
	**Water Activity**	0.78	0.76	0.75	0.74	0.76	0.71	0.71														
	**Firmness (g)**	778.74	788.16	786.04	764.6	898.98	797.1	749.43	851.93	853.32	808.41	751.97	849.94	945.47	761.31	827.39	723	699.52	738.08	827.68	853.37	828.48
**Natural-High**	**Springiness (%)**	91.4	93.01	91.4	92.33	91.46	91.03	91.12	90.88	90.92	90.63	90.35	91.68	91.82	90.74	90.82	92.24	92.8	92.92	92.77	90.67	90.22
	**Water Activity**	0.76	0.74	0.72	0.73	0.73	0.67	0.67														

Supplementary Table S3. Color data

Formulation	Measurement		
	L*	a*	b*
**Artificial-Low**	18.89	1.1	7.66
	19.08	1.03	7.42
	19.64	1.16	7.67
**Natural-Low**	19.04	0.24	7.49
	19.34	0.24	8.7
	20.95	0.09	8.37
**Artificial-High**	13.17	6.35	13.61
	13.3	6.59	13.97
	13.86	6.13	13.17
**Natural-High**	14.47	6.74	14.09
	14.64	6.82	14.01
	14.74	7.03	14.29

Supplementary Table S4. Total phenolic content data

Dose Level (mg extract/mL)	Measurement (µg GAE/mL extract)	(µg GAE/mL extract)	(µg GAE/mL extract)
0.5	126.0734	141.0452	126.9209
1.0	234.2655	227.2034	222.6836
2.0	428.6158	428.0508	433.9831
4.0	737.0904	739.0678	763.0791
8.0	1252.345	1242.74	1282.006

Supplementary Table S5. Two-way analysis of variance results for effects of colorant source, colorant concentration, and their interaction on physical properties of gummy candies

Variables		P-value		Effect size	
**Water activity**			<0.0001		0.597
	**Source**	0.0274		0.093	
	**Concentration**	<0.0001		0.504	
	**interaction**	0.8973		<0.001	
**Firmness**			0.0006		0.192
	**Source**	0.1384		0.023	
	**Concentration**	0.0975		0.028	
	**interaction**	0.0003		0.142	
**Springiness**			<0.0001		0.235
	**Source**	<0.0001		0.215	
	**Concentration**	0.2476		0.013	
	**interaction**	0.3893		0.007	
**L* (color)**			<0.0001		0.971

	**Source**	0.0319		0.024	
	**Concentration**	<0.0001		0.944	
	**interaction**	0.3989		0.003	
**a* (color)**			<0.0001		0.998
	**Source**	0.0469		0.001	
	**Concentration**	<0.0001		0.983	
	**interaction**	<0.0001		0.014	
**b* (color)**			<0.0001		0.989
	**Source**	0.0322		0.009	
	**Concentration**	<0.0001		0.980	
	**interaction**	0.9017		<0.001	
